# Environmental marine pathogen isolation using mesocosm culture of sharpsnout seabream: striking genomic and morphological features of novel *Endozoicomonas* sp.

**DOI:** 10.1038/srep17609

**Published:** 2015-12-07

**Authors:** Pantelis Katharios, Helena M. B. Seth-Smith, Alexander Fehr, José M. Mateos, Weihong Qi, Denis Richter, Lisbeth Nufer, Maja Ruetten, Maricruz Guevara Soto, Urs Ziegler, Nicholas R Thomson, Ralph Schlapbach, Lloyd Vaughan

**Affiliations:** 1 Institute of Marine Biology, Biotechnology and Aquaculture, Hellenic Center for Marine Research, Heraklion, Crete, Greece; 2 Functional Genomics Center Zürich, University of Zürich, Switzerland; 3 Institute for Veterinary Pathology, Vetsuisse Faculty, University of Zürich, Switzerland; 4 Center for Microscopy and Image Analysis, University of Zürich, Switzerland; 5 Center for Fish and Wild Animal Medicine, Vetsuisse Faculty, University of Bern, Switzerland; 6 The Wellcome Trust Sanger Institute, Hinxton, Cambridge, United Kingdom

## Abstract

Aquaculture is a burgeoning industry, requiring diversification into new farmed species, which are often at risk from infectious disease. We used a mesocosm technique to investigate the susceptibility of sharpsnout seabream (*Diplodus puntazzo*) larvae to potential environmental pathogens in seawater compared to control borehole water. Fish exposed to seawater succumbed to epitheliocystis from 21 days post hatching, causing mortality in a quarter of the hosts. The pathogen responsible was not chlamydial, as is often found in epitheliocystis, but a novel species of the γ-proteobacterial genus *Endozoicomonas*. Detailed characterisation of this pathogen within the infectious lesions using high resolution fluorescent and electron microscopy showed densely packed rod shaped bacteria. A draft genome sequence of this uncultured bacterium was obtained from preserved material. Comparison with the genome of the *Endozoicomonas elysicola* type strain shows that the genome of *Ca*. Endozoicomonas cretensis is undergoing decay through loss of functional genes and insertion sequence expansion, often indicative of adaptation to a new niche or restriction to an alternative lifestyle. These results demonstrate the advantage of mesocosm studies for investigating the effect of environmental bacteria on susceptible hosts and provide an important insight into the genome dynamics of a novel fish pathogen.

The aquaculture industry is constantly expanding to meet the needs of the global population and ever growing demand for quality protein. Economic sustainability of this industry requires an increasing diversification of cultured fish species. A prerequisite of the introduction of new species to aquaculture is the development of larval cultures and assessing their growth and health on exposure to water from the open sea. The semi-intensive “mesocosm” technique can be used to determine the specific biological, ecological and nutritional needs of each species, as rearing methodologies used in other established species may not be applicable^1^. Under the mesocosm technique, unfiltered sea water is supplied to tanks in order to enhance natural planktonic productivity, providing live feed prey to the fish larvae in addition to supplements of cultured live feeds such as rotifers and *Artemia*^2^,^3^. A major threat to this type of rearing system is the introduction of pathogenic microorganisms from sea water leading to the emergence of novel diseases in potentially vulnerable hosts. Marine microbial pathogens may disperse either freely in the water column or via intermediate eukaryotic hosts. While bacterial pathogens are largely in equilibrium with host defences in wild fish populations^4^, they are likely to have more severe effects on farmed animals, where the conditions can lead to overcrowding and host stress^5^.

Epitheliocystis is a serious disease of freshwater and marine fish, found throughout the world affecting many different species, both wild and cultured^6^,^7^,^8^. The characteristic cysts are found in the gills of juvenile and older fish, whereas larvae also exhibit skin lesions. The larvae suffer the greatest mortalities, with complete cohort losses not uncommon^9^.

The primary microbial culprits were long thought to be members of the phylum Chlamydiae^6^, a view strengthened by the molecular identification of *Candidatus* Clavochlamydia salmonicola and *Candidatus* Piscichlamydia salmonis, endemic within wild salmonid and trout populations of the genus *Salmo* in Northern hemisphere marine and freshwater environments^10^,^11^,^12^,^13^. Within the past few years, relatives of *Ca.* P. salmonica have been identified in marine vertebrates from the Southern hemisphere waters of Australia^14^,^15^,^16^, from aquacultured fish out of Africa^17^ and from cleaner wrasse in contact with farmed salmon in Norway^18^. The chlamydial family *Simkaniaceae* is also represented, with *Ca*. Syngnamydia venezia identified in the broad nosed pipefish, *Syngnathus typhle*^19^ and *Ca*. Syngnamydia salmonis in Atlantic salmon, *Salmo salar*^20^. However, Chlamydiae are not the only bacterial agents identified in association with epitheliocystis, as recent studies have shown that β- and γ-proteobacteria^21^,^22^,^23^ (Seth-Smith, *ISME J*, in press) also play a role.

A great hindrance to progress in combating the disease is the lack of an experimental setup to follow infections, exacerbated by our inability to successfully cultivate the epitheliocystis agents. We used a semi-intensive mesocosm system to culture sharpsnout seabream (*Diplodus puntazzo*) larvae in natural sea water, mimicking the natural environment^1^,^2^,^3^. This species is of interest to the aquaculture industry and has previously shown to be susceptible to epitheliocystis outbreaks^9^. We observed the larvae over several weeks through the development and resolution of an epitheliocystis infection. We have applied new techniques to investigate the bacteria responsible, resulting in the thorough morphological, molecular and genomic description of the novel γ-proteobacterium *Candidatus* Endozoicomonas cretensis. This strategy could be widely adapted to different hosts and diseases, giving information on cultured and uncultured agents alike, identifying further emerging pathogens, and providing a key to their genomic analysis.

## Results

### Mesocosm epitheliocystis infection model

We sought to develop a system to investigate epitheliocystis-causing bacteria in the eastern Mediterranean, especially important given the lack of methods for culture of epitheliocystis agents *in vitro*. Using large scale mesocosm tank facilities at Hellenic Centre for Marine Research (HCMR) in Crete (Figure S1), we exposed larvae of a susceptible host species (*Diplodus puntazzo*) to natural sea water in an experimental tank, with control larvae cultured in a second tank supplied with saline water from a deep borehole supply. Larvae were monitored every 1–4 days for behaviour and morphologically for overt disease as well as molecularly for chlamydial epitheliocystis agents from 9 days post hatching (dph). A series of filtered plankton fractions from the tank water was collected at 13 dph to determine whether potential epitheliocystis agents were free in the water column or associated with particular fractions.

**Figure 1:**
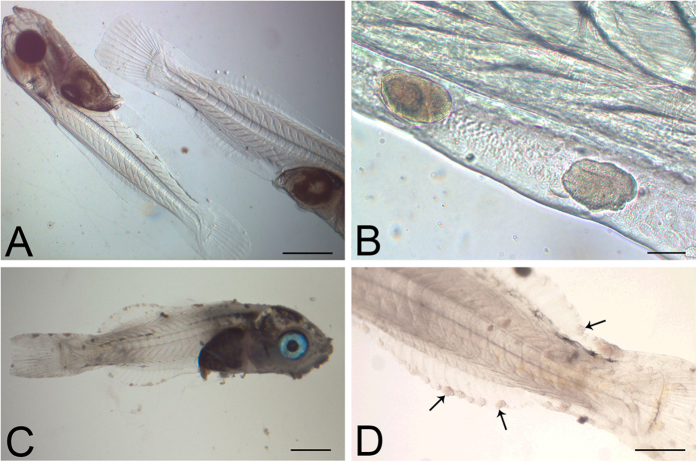
Bright field images of representative whole larvae. (**A**,**B**) Larvae 21 dph showing epitheliocystis infection, the cysts most readily apparent in the periphery of the fins. (**C**,**D**) Larvae 28 dph, heavily infected with epitheliocystis affecting fins and skin. Several cysts are indicated with arrows. Images were taken immediately after larval euthanasia. Scale bars represent: (**A**,**C**) 1 mm, (**B**) 50 μm, (**D**) 500 μm.

The first epitheliocystis lesions were detected in the fins of larvae from the experimental tank at 21 dph (Fig. 1A,B), and first positive PCR signal for chlamydial signature sequence in DNA extracted from affected larvae. The severity of the epitheliocystis infections increased over the next 7 days, affecting all larvae screened, with the 28 dph larvae covered in multiple cysts (Fig. 1C,D). By 35 dph the infections were decreasing and only a few infected larvae detected. No epitheliocystis was observed in larvae from the control borehole water tank at any stage, neither was the chlamydial signature sequence detected in sacrificed control larvae. From the experimental mesocosm, 19.6% of the larvae survived to the juvenile stage (55 dph), compared with 53.3% survival in the control tank (p < 0.000). The growth rates (Figure S2; p = 0.348) and final weights of surviving fish at 69 dph (experimental larval mean of 0.27 g compared to control larval mean of 0.22 g; p = 0.107) were not significantly different between the two tanks.

### Pathology of infected larvae

**Figure 2:**
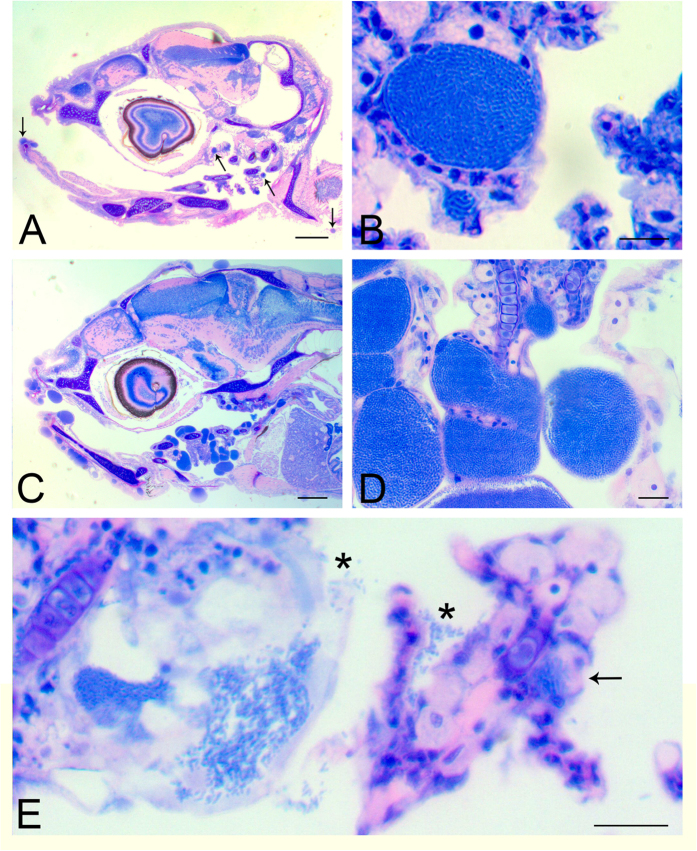
Histopathology of representative infected larvae. (**A)** Section of the head of a 21 dph larva with cysts (arrows) in gills and skin. (**B)** A typical large cyst in the gills of a 21 dph infected larva with particulate appearance, and a small one below it, are visible. (**C**) Section of the head of an infected larva 28 dph, with extensive and multiple cysts in the epithelium of the skin, mouth and gills. (**D**) Higher magnification of the gill lamellae from (**C**) showing bacterial-sized particles within cysts. (**E**) An empty inclusion from a 21 dph larva with freed bacteria (*) attaching to the next filament. There is also a newly formed small inclusion (arrow) at the base of the lamella. Scale bars represent: (**A**,**C**) 200 μm, (**B**) 10 μm, (**D**,**E**) 20 μm.

Histological examination of sections of 21 dph larvae reveals large cysts surrounded by the cytoplasm of epithelial cells and a hyaline eosinophilic membrane (Fig. 2). These cysts are associated with changes to the skin or gill epithelium with very mild hyperplasia of the epidermal or gill epithelium. The cysts appear to be the result of displacement of the cytoplasm and nucleus by moderately sized (10–30 μm diameter), well-marginated vacuoles containing basophilic granular material (Fig. 2A,B). At the height of the infection severity (28 dph), multiple cysts of 20–100 μm diameter are found, often closely spaced, richly populating skin and gill epithelia (Fig. 2C,D). Occasionally, empty cysts can be seen, with liberated bacteria either free or adhering to adjacent tissue (Fig. 2E), inferring a possible mode of reinfection.

### Ultrastructural studies of epitheliocysts

**Figure 3:**
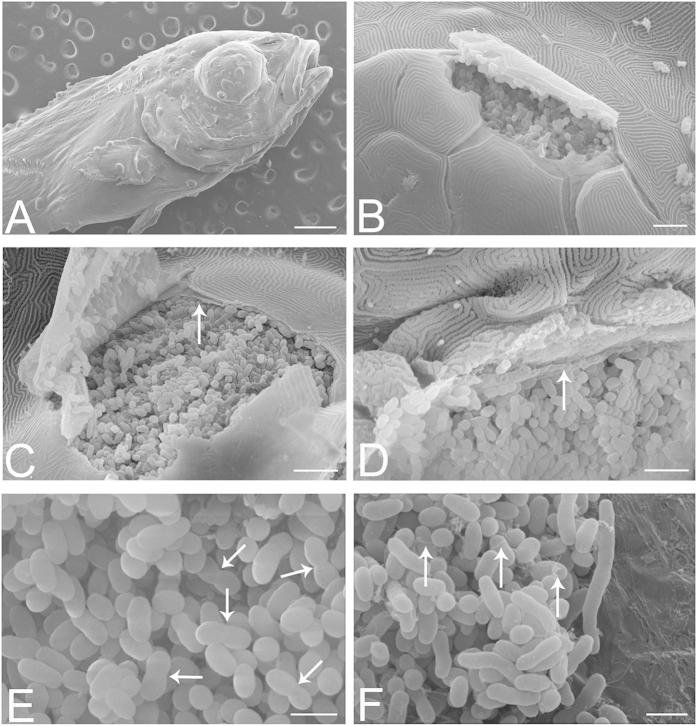
SEM of infected larvae at 21 dph. (**A**) Surface view of larva, showing cysts on skin and fins. (**B**) A ruptured cyst beneath the skin appears to be densely packed with bacteria. (**C**) Ruptured cyst revealing the bacteria to be separated from the epithelial cells by a membrane (arrow) with a fibrous-like appearance, and with multiple fenestrations or perforations. Should the cyst microenvironment be tightly regulated by the enveloping cells, then presumably these would need to form tight junctional contacts with each other and with the cyst itself. (**D**) Bacteria appear to be in intimate contact with the surrounding membrane (arrow), which appears to partially fold around the bacteria, creating shallow bays for them. (**E**) Within a cyst, bacteria appear to be dividing (arrows). (**F**) Another view shows elongated bacterial forms and filaments (arrows). Scale bars represent: (**A**) 500 μm, (**B**) 10 μm, (**C**,**D**) 5 μm, (**E**,**F**) 2 μm.

Electron microscopy (EM) was used to further characterise these bacteria in the absence of a cultured strain. Initial Scanning Electron Microscopy (SEM) performed on larvae at 21 dph was used to assess the external aspects of the larval cysts in the skin, confirming the external morphology of the cysts (Fig. 3A), and providing views of the interior of cysts ruptured during the preparation process (Fig. 3B–F). The cysts appear to be fully enclosed by a surrounding membrane, itself embedded below a layer of epithelial cells (Fig. 3C,D). Within the cysts, densely packed rod shaped bacteria can be seen, some of which appear to be dividing (Fig. 3E), as well as longer bacterial forms and a network of thin filaments (Fig. 3F).

**Figure 4:**
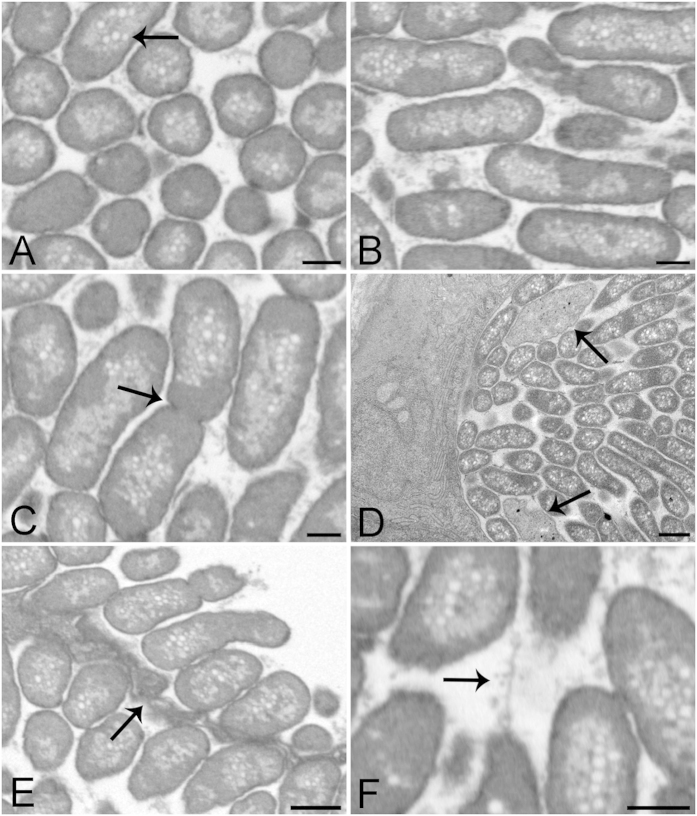
Representative FIB-SEM and TEM images of epitheliocysts from larvae 24 dph and 20 dph respectively. **(A**,**B**) FIB-SEM images from a cyst 3D volume at cross and longitudinal planes showing bacterial size, orientation, shape and compactness. Bacteria are surrounded by a densely stained single membrane containing a dense homogeneous layer. The centres of the bacteria appear lighter with many pale vacuoles (arrow). (**C**) FIB-SEM image of dividing bacterium (arrow). Granular material is visible between the bacteria. (**D**) TEM image of a transverse section of 20 dph larva showing the edge of a cyst with bacteria cut lengthwise and in cross-section. Two amorphous bodies are also present (arrows). (**E**) FIB-SEM of two neighbouring cysts separated by a cyst boundary (arrow). This tissue presents involuted membranes, partially enveloping the bacteria. (**F**) FIB-SEM image showing a network of thin filaments between the bacteria. Scale bars represent: (**A**–**C**,**F**) 500 nm, (**D**,**E**) 1 μm.

Further imaging using Transmission Electron Microscopy (TEM) and 3D datasets from Focused Ion Beam-Scanning Electron Microscopy (FIB-SEM) allowed an accurate identification of bacterial shape within the cyst, and was used to show that they are rod shaped with a diameter of approximately 0.8 μm and length of 2.5 μm (mean of 5 measurements, Fig. 4). Throughout the cysts, the bacteria appear densely packed and homogeneous (Fig. 4A), as in SEM (Fig. 3C), not displaying a typical chlamydial lifecycle which would show dividing reticular bodies and more dense infectious or elementary bodies towards the middle of the cyst^13^. The bacteria often orient in the same direction, producing 2D images of the circular diameter in one plane and elongated cross-sections in other planes (Fig. 4A,B). Dividing bacteria can be seen (Fig. 4C and video S1), in addition to elongated forms and amorphous bodies (Fig. 4D). There is no obvious condensed nuclear material within the bacteria, but many pale vacuoles which may be lipid-containing (Fig. 4A and Video S1). The interior of the cyst appears granular or particulate (Fig. 4), and the membrane surrounding the cyst appears to fold around the enclosed bacteria (Fig. 4E). In between the bacteria we observe numerous thin filaments (Fig. 4F), forming a network around the bacteria. Although cysts grow in size with infection duration, no significant differences in the morphology of cysts or the bacteria within was seen in TEM from 20, 21, 24 or 28 dph.

### Identification of the pathogen responsible

Identification of the infectious agents within the cysts was attempted initially using Chlamydiae-specific 16S rRNA gene primers. Results were inconsistent, with a diversity of chlamydial sequences obtained from the larvae, the plankton fractions and flow through water (Supplementary information and Figure S3). A series of fluorescent *in situ* hybridisation (FISH) probes designed to locate specific sequences to the cysts provided uninformative results, with no strong signal detected from the cysts or elsewhere in larval sections (Figure S4).

**Figure 5:**
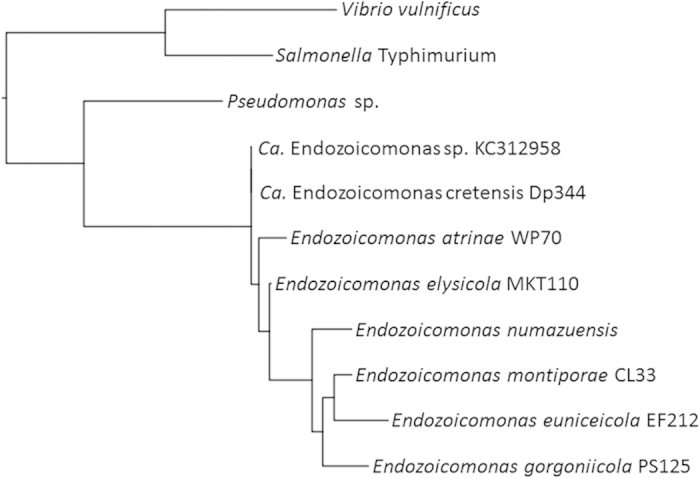
Phylogenetic analysis of the novel epitheliocystis agent within the γ-proteobacteria. Representative 16S rRNA gene sequence Dp344 amplified from 21 dph larva, compared against all *Endozoicomonas* species, contextualised with other γ-proteobacteria. Sequences aligned with muscle, phylogeny by PhyML. Bootstraps within the *Endozoicomonas* genus are all over 85%. A further nine sequenced clones showed 1-2 mismatches relative to Dp344 over the length of the 16S rRNA gene (data not shown).

In order to screen for more diverse pathogens, universal 16S rRNA gene amplification was performed on DNA from 21 dph and 24 dph larval samples. The majority of amplicons sequenced had top BLAST hits against *Endozoicomonas* 16S rRNA gene sequences, a recently described γ-proteobacterial genus. The *Endozoicomonas* sp. 16S rRNA gene sequences from the sharpsnout seabream larvae are identical to that from an epitheliocystis outbreak in Colombia in cobia larvae (Fig. 5). Comparing within the genus, this sequence has 99.1% (1425/1437 bp) identity to *E. elysicola* and 98.5% (1444/1465 bp) identity to *Endozoicomonas atrinae*.

**Figure 6:**
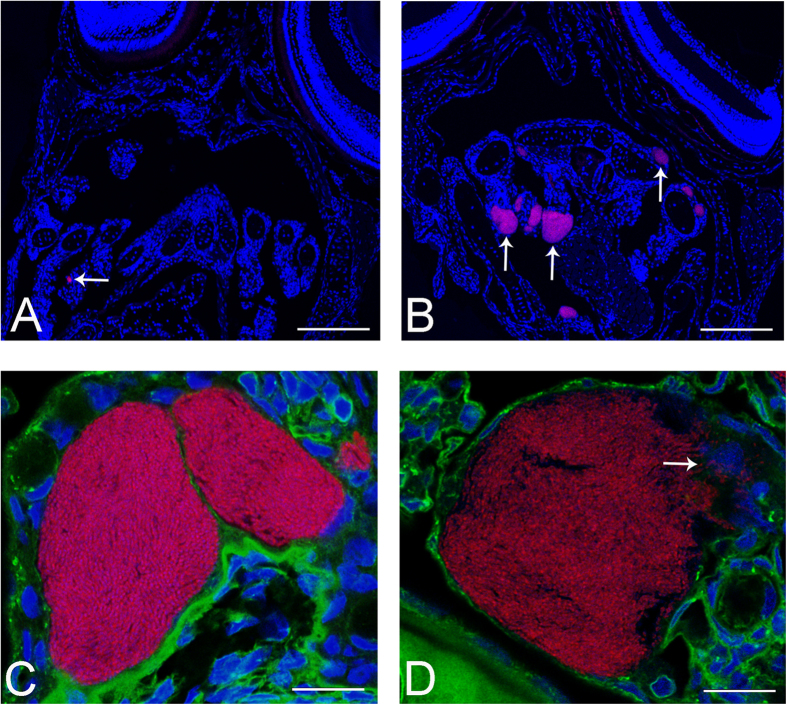
FISH of larvae using probes based on Endozoicomonas 16S rRNA gene sequences. All sections are hybridised with Endo-0474-Cy3 (red) for bacteria and are counterstained with DAPI. (**A**) Section of larva 20 dph with one bacteria-containing cyst visible (arrow). (**B**) Section of larva 24 dph with several cysts in the gills visible (arrows). Sections from 28 dph contained many more labelled cysts (data not shown). (**C**,**D**) High resolution confocal images (deconvolved) of *Endozoicomonas* epitheliocysts from larvae 24 dph additionally labelled with concanavalin A-Alexa488, showing large, granular bacteria-containing cysts lying within the gill epithelium, displacing the nuclei of surrounding cells. Arrow (**D**) indicates several macrophages attacking the bacteria where the cyst is ruptured. Scale bars represent: (**A**,**B**) 100 μm, (**C**,**D**) 10 μm.

Confirming that this bacterium is responsible for the pathology observed, FISH using probes designed against the *Endozoicomonas* sp. produced clearly labelled cysts (Fig. 6A,B and Figure S4), whose locations correlate with those seen in adjacent Haematoxylin-Eosin (HE) stained sections. Thus the bacteria within the cysts are a novel pathogenic *Endozoicomonas* species. High resolution imaging with additional Concanavalin A (Fig. 6C,D) clearly shows bacteria-containing cysts displacing epithelial cell cytoplasm, but clear cell borders cannot be made out to determine the intracellular nature of the cysts. In some cases macrophages are seen invading ruptured cysts (Fig. 6D).

Using a quantitative PCR (qPCR) system targeting the novel *Endozoicomonas* bacteria, extracts from 21 dph and 24 dph larvae, as well as from serial fractions from both tanks, were assayed to determine the load of *Endozoicomonas* in the larvae, with chlamydial quantification for comparison. The results indicate that the mean pathogen load for a larva 21 dph is 17 860 000 16S rRNA gene copy number equivalents, which increases to 113 066 667 at 24 dph (Table S3). *Endozoicomonas* was not found in any of the filtered water fractions from either the experimental or control tank at 13 dph. Thus we have no evidence that this pathogen uses any organism within the plankton fractions as an intermediate host. Signal from chlamydial-specific DNA was found in all samples, indicating that these may be ubiquitous environmental organisms. Chlamydial loads within infected larvae were 600–8500 times lower than loads of *Endozoicomonas*, and did not increase over the course of the infection.

### Genome sequencing directly from infected material

A draft genome of the novel *Endozoicomonas* epitheliocystis agent was generated from preserved infected material. DNA extracts from larvae and micromanipulated cysts from 21 dph and 28 dph were sequenced, with three samples giving data which mapped to the *Endozoicomonas* type strain reference genome (Table S4), confirming the epitheliocystis pathogen identity. Sample Dpd28tailN from 28 dph had the highest coverage (63×; Table S4), and was chosen for further analysis, producing an optimised assembly of 5.79 Mb over 602 scaffolds. This may not represent the entire genome as small scaffolds were discarded (see Methods), and a complete genome would require the bacterium to be in culture.

The genome of the novel pathogen is clearly distinct from that of the *E. elysicola* type strain DSM 22380^24^, isolated from the gastrointestinal tract of a sea slug^25^. The assembly of Dpd28tailN maps to only three-quarters (4.2 Mb) of the reference genome and contains over 1 Mb of assembled sequence which does not match the reference (Figure S5). Given this large difference in accessory DNA, we assessed whether this new pathogen of sharpsnout seabream constitutes a new species of *Endozoicomonas*. The new pathogen shares an average nucleotide identity (ANI) with *E. elysicola* of 95.8%, over 53.6% conserved DNA, compared against the species definition of 95% ANI over 69% conserved DNA^26^. Furthermore, the new pathogen shares only 49.6% of conserved protein coding genes with *E. elysicola*, far below the species cut-off level of 85%^26^. Given this analysis, and the differences in phenotype and host between the two strains, we propose this to be a new species, designated *Candidatus* Endozoicomonas cretensis, due to its occurrence in Crete.

A preliminary comparison of the genome of *Ca*. E. cretensis genome against that of the *E. elysicola* type strain indicates that there has been an expansion of Insertion Sequences (ISs) since their most recent common ancestor. Thorough manual annotation indicates that there are 790 CDSs annotated as transposases or integrases from a total of approximately 5840 annotated CDSs (14%). In contrast, only 2 CDSs are annotated as transposases within the *E. elysicola* genome. In particular, nine novel ISs have been identified as having expanded within the *Ca*. E. cretensis genome, with estimated copy numbers from 11 to over 60. The vast majority of these occur at scaffold ends and contribute to the fragmentation of the assembly. These insertions have led to gene disruption, with 60 pseudogenes identified adjacent to IS sites. In addition there are over 250 annotated transposases which appear not to have expanded, of which over 150 are themselves pseudogenes.

Functional gene loss has also occurred without the effect of ISs. Many frameshifts and premature stop codons can be identified within the annotation, leading to the predicted inactivation of over 100 genes. Thus the genome of *Ca*. E. cretensis is undergoing degradation, possibly as a result of having passed through a population bottleneck, which may now be restricting the metabolic repertoire of the bacterium.

Further differences between the genome of *Ca.* E. cretensis and *E. elysicola* include the insertion of two phages, the larger of which is over 60 kb. Most of the cargo genes encode uncharacterised proteins, in addition to genes encoding a toxin antitoxin system and a Type I restriction modification system. Also of note within the accessory scaffolds is a putative non-ribosomal peptide synthetase gene of 8 kb. Further annotation and analysis will be published separately, investigating possible links between accessory genes, inactivated genes and the lifestyle of this strain as compared to other species of *Endozoicomonas*.

## Discussion

We present the first example of epitheliocystis investigated under controlled conditions. Using the susceptible sharpsnout seabream host larvae in a sea water mesocosm, epitheliocystis lesions were seen to develop, cause mortality, and resolve over a time period of 20–35 dph. While mortality is often high in such larval cultures^27^, epitheliocystis was found to be a significant burden on the health of these fish. Given these findings, we will continue with our plans to test further fish species at HCMR Aqualabs to refine this novel approach and our analytical procedures.

The most common causes of epitheliocystis infections are chlamydial agents. However, in this study based in Crete, a novel species of epitheliocystis-causing pathogen has been identified as a member of the γ-proteobacteria and named *Ca*. Endozoicomonas cretensis. Many species within this genus have been identified over the past few years, and are mostly characterised as symbionts of marine organisms, including sea slugs, comb pen shell and corals^25^,^28^,^29^,^30^. Contrary to their role in this study as pathogens, it has been suggested that related species may be indicators of coral health^31^. Recently *Endozoicomonaceae* have been confirmed as common members of the whole coral microbial community although not within the symbiotic coral core microbiome^32^. Further and more detailed investigations are required to determine the varying features of this genus, and the approach we describe here may prove invaluable in this pursuit. The most closely related organism to *Ca*. E. cretensis identified to date also causes epitheliocystis in cobia larvae in Colombia^22^. Therefore this species may be a pathogen of fish larvae with a global reach. Several species of this marine genus have been cultured, indicating that they are not obligate intracellular bacteria. This knowledge may aid attempts to culture the first epitheliocystis agent.

Investigations into the lifestyle of these bacteria have produced some phenotyping results^25^,^29^,^30^ and antibiotic resistance profiles^29^. Our study has extended this knowledge with a highly detailed ultrastructural morphological study of the bacteria and cysts in which they reside, in concert with genomic sequence analysis. *Ca*. E. cretensis is likely to have divergent phenotypes from these other strains given the level of pseudogene formation observed.

As this pathogen of sharpsnout seabream is as yet uncultured, the best way to generate information on its lifestyle is through careful genomic analysis. While reference genomes for three *Endozoicomonas* species have been published^24^, detailed manual annotation and analysis of metabolic pathways is required to gain the best insight, and will be presented for *Ca*. E. cretensis in a subsequent publication. Preliminary analysis indicates that this bacterium is undergoing genomic decay through massive IS expansion and mutational inactivation of genes, a process which has previously been observed in pathogens adapting to niche change or host restriction^33^,^34^,^35^,^36^. Given the wide host range of the *Endozoicomonaceae*, it will be fascinating to investigate the molecular basis for their ability to colonise diverse niches.

The production of a draft genome from preserved infected material represents a significant advance in the scope of molecular methods available for the study of diseases such as epitheliocystis. The suite of techniques presented here: pathology, microscopy, molecular analysis and genome sequencing, demonstrate how it is possible to concretely identify and investigate novel pathogens from infected material stored in formalin and RNALater. Culture of infectious agents remains the best way of obtaining complete genomes, and is essential to perform infection studies, antibiotic sensitivity tests and to meet Koch’s postulates. Isolation of *Ca.* E. cretensis should be attempted on marine agar, although a host may be required for growth given the degree of genome degradation; in this case, various approaches can be taken including possible culture in fish cell lines^37^, developing a fish model such as zebrafish, or a non-model marine host such as the sharpsnout seabream. However, in many cases it is impractical or impossible to achieve, due to sampling, contamination or culture condition issues, and we illustrate how valuable data can nonetheless be obtained in these situations.

Epitheliocystis remains a persistent infection in fish and a significant challenge to aquaculture. We have extended the knowledge on the diversity of pathogens known to cause this disease, and demonstrate that larval cultures in the Mediterranean are highly susceptible. Further knowledge on the diversity, distribution, source and lifestyle of these pathogens is key to devising methods for the identification, management and treatment of aquaculture species in order to optimise their welfare.

## Methods

### Larval culture

Sharpsnout seabream were reared in the facilities of the Institute of Marine Biology, Biotechnology and Aquaculture, HCMR, Crete, using semi-intensive mesocosm technology^3^. Two 40 m^3^ tanks (Figure S1) were filled with either natural sea water (salinity 40 practical salinity units; psu) or sea water from a 200 m deep borehole (salinity 34 psu) and each stocked with approximately 200 000 sharpsnout seabream eggs. Water was renewed at an increasing rate from 5% daily at the beginning of the experiment to 50% daily at 25 dph. The fish were fed on enriched rotifers, *Brachionus plicatilis*, from 2 dph and enriched *Artemia* sp. nauplii from 13 dph and dry food from 22 dph, while microalgae *Chlorella minutissima* were added daily in the rearing water from 2 dph (Figure S2). Abiotic factors of the two tank cultures were measured: tanks were found to be equivalent environments in terms of temperature, oxygen content and pH. Larval growth was assessed by analysing 10–15 fish every 1–4 days for length, with the growth in each tank compared using regression analysis^38^. Final weight of the fish was determined at 69 dph with over 40 fish assessed, analysed by t-test. The survival of larvae cannot be determined until the fish are moved from the mesocosm tanks to on-growing tanks of 10 m^3^ and can be counted: this was performed at 55 dph and analysed statistically using Chi squared.

### Sample collection

Fish larvae (10–20) were assessed every 1–4 days from 9 to 28 dph and visualised using a Nikon SMZ800 dissecting microscope. Epitheliocystis was initially diagnosed through the presence of visible cysts on skin and fins of larvae. Fish were euthanized in buffered 3-aminobenzoic acid ethyl ester in filtered seawater and whole larvae or microdissected cysts were immediately transferred to storage solutions at 4 °C: 10% buffered formalin in seawater or 4% formaldehyde: 1% glutaraldehyde (4F:1G) fixative^9^ for 24 h for histopathological analysis; 2.5% glutaraldehyde in 0.1 M sodium phosphate buffer, pH 7.5 for electron microscopy; 70% ethanol or RNALater for DNA analysis (Figure S2).

Tank water was sampled at 13 dph in 10 litre volumes, and plankton fractions collected by sequential filtering through a series of nylon net filters with pore sizes 250 μm, 120 μm, 50 μm, 25 μm and 10 μm and backwashing the filter with sterile filtered sea water. Material in the flow through (<10 μm) was pelleted.

### Ethics statement

Ethics approval of implementation of research with fish for Pantelis Katharios was forthcoming from the Veterinary Office of Heraklion (Approval number 6329). Ethics approval was additionally forthcoming from the Ethics Advisor of Aquaexcel (Application 01/05/15/004/B), who stated that “The Ethics Advisor did not identify any problems with the proposal. The sampling of fish from within existing populations within the facility was performed in accordance with the approved guidelines and the humane killing proposed meet 3R requirements in respect of reduction and refinement”.

### Histopathology

Samples fixed in 4F:1G were dehydrated in an ethanol series and embedded in glycol methacrylate resin^9^. Serial 3–5 μm sections were stained with methylene blue/azure II/basic fuchsin^9^, Gram stain and Giemsa and examined under a light microscope (Leica DMLB) for diagnosis of epitheliocystis. Microphotographs were taken with an ALTRA 20 digital camera and analysed using ImagePro 6.1 (MediaCybernetics).

Additionally, fixed larvae were sectioned into three parts, aligned vertically in a cube of boiled egg-white, briefly fixed again in neutrally buffered 4% formalin, dehydrated in an ethanol series, followed by Xylol and paraffin embedding (formalin fixed paraffin embedded, FFPE). Serial cross-sections of larvae (3 μm) were stained with HE using standard procedures, or processed for FISH.

### DNA extraction

DNA was extracted from larvae, individual micromanipulated cysts or plankton fractions using standard phenol:chloroform protocols or DNeasy Blood and Tissue kit (Qiagen, Hilden, Germany).

### TEM

Fish larvae fixed with 2.5% glutaraldehyde in 0.1 M sodium phosphate buffer, pH 7.5 at 4 °C were embedded into epoxy resin and prepared for TEM according to standard procedures^13^. Gill sections containing epitheliocystis lesions were selected from epoxy resin blocks using semithin sections (1 μm) stained with toluidine blue (Sigma–Aldrich). Ultrathin sections (80 nm) were mounted on copper grids (Merck Eurolab AG, Dietlikon, Switzerland), contrasted with uranyl acetate dihydrate (Sigma–Aldrich) and lead citrate (Merck Eurolab AG) and investigated using a Philips CM10 transmission electron microscope. Images were processed with Imaris 7.6.1 (Bitplane, Oxford Instruments) and assembled for publication using Photoshop CS4 extended, version 11.0.2 or CS6 extended, version 13.0 × 32 (Adobe).

### SEM

Samples for SEM were fixed in 2.5% glutaraldehyde in 0.2 M sodium cacodylate buffer pH 7.4, washed with 0.1 M sodium cacodylate buffer pH 7.4, post-fixed with 1% osmium tetroxide and dehydrated in an ascending alcohol series, mounted on stubs and sputter-coated with gold-palladium. Fish larvae were viewed using a JEOL JSM-6390LV scanning electronic microscope at 15 kV at the Electron Microscopy Laboratory of the University of Crete.

### FIB-SEM

Sample blocks were fixed in 2.5% glutaraldehyde in 0.1 M sodium cacodylate buffer pH 7.4, followed by post-fixation in 1% osmium tetroxide and contrasted with 2% aqueous uranyl acetate. Samples were dehydrated in an ethanol series, followed by propylene oxide and embedded in Epon 812 resin. Semithin and ultrathin sections were obtained to identify the cysts in the blocks, which were attached to 12 mm stubs by conductive carbon cement followed by carbon coating. 3D datasets were acquired with an Auriga 40 Crossbeam (Zeiss, Oberkochen, Germany) using the FIBICS Nano patterning engine (Fibics Inc, Ottawa, Canada). The gallium-ion beam for milling was used at 30 kV, 600 pA current and the images were acquired at an acceleration voltage of 1.5 kV using an in-lens energy selective backscattered electron detector (ESB) with a grid voltage of 1.3 kV. The pixel size was set to 5 nm and the milling depth to 15 nm. The image stacks were aligned with TrackEM2^39^, segmented with Ilastik, 1.1^40^, and the aligned dataset was visualized with Imaris.

### Bacterial identification

The presence of chlamydial DNA was determined by PCR targeting the Chlamydiales-specific 280 bp 16S rRNA gene signature sequence^41^. Subsequent bacterial identification used 16S rRNA genes amplified using chlamydial-specific systems 16SigF and SB1^10^, EcF1: 5′-ATTGAATGCTGACGGCGTGG-3′ and CHR11: 5′-CGCCATTACTAGCAATTCC-3′, and a universal bacterial PCR system fD1: 5′-AGAGTTTGATCCTGGCTCAG-3′ and rD1: 5′-AAGGAGGTGATCCAGCC-3′ 42. Negative controls (dH_2_O) were performed in triplicate, with *Waddlia chondrophila* DNA as the positive control. Freshly amplified products were cloned into pCR2.1-TOPO (Invitrogen, Basel, Switzerland) and individual clones (up to 10 from each larva) sequenced (Microsynth, Switzerland). Resulting reads were quality checked, assembled and edited with CLC Main Workbench, version 7.02 (CLC bio, Aarhus, Denmark). Sequences were screened against the GenBank database^43^, aligned using MUSCLE^44^ and phylogenies were generated using PhyML with 100 bootstraps, both within SeaView^45^. The 16S rRNA gene sequence of *Candidatus* Endozoicomonas cretensis has been deposited with EMBL under accession number LN626318.

### FISH

FISH was performed using the Ventana Discovery XT automated platform, with automated deparaffinisation and pretreatment with 0.2 M HCl in PBS followed by pepsin (500 μg/ml in this buffer) for 4 min. Following addition of 50 ng probe per slide in a hybridisation buffer comprising 6× saline-sodium citrate buffer (SSC), 5 × Denhardt’s solution and 12% dextran sulphate, samples were denatured at 90 °C for 4 min and hybridised at 48 °C overnight. A wash with 2×SSC at 48 °C was followed by manual post-staining with 4′-6-diamin-2-phenylindole (DAPI) at 10 μg/ml for 5–10 minutes to visualise bacterial and host DNA. Concanavalin A-AlexaFluor488 (25 μg/ml) (Life Technologies) was added for 10–30 min to visualise glycoprotein-containing membranes and structures.

A variety of control and test probes were designed against relevant 16S rRNA gene sequences to be 15–30 nucleotides in length, 50–70% G + C, with a ΔG below −13 (determined using http://131.130.66.200/cgi-bin/probecheck/probecheck.pl)^46^ (Table 1).

Overviews of the sections were obtained using a Hamamatsu Nanozoomer 2.0 HT with bright field and fluorescence modules (4-band filter set, optimized for DAPI/FITC/Cy3.5/Cy5). Higher resolution images were prepared using a confocal laser scanning microscope (CLSM, Leica TCS SP5, Leica Microsystems) with the 561 nm, 488 nm and 405 nm laser lines sequentially. 3D image stacks were collected sequentially according to Nyquist criteria and deconvolved using HuygensPro via the Huygens Remote Manager v2.1.2 (SVI, Netherlands)^47^. Images were prepared for publication using Imaris.

### qPCR

In addition to the *Chlamydiales* specific qPCR system^48^, a *Ca*. E. cretensis-specific Taqman system was designed, according to the restrictions that amplicons should be optimally 150 bp, the primers 30–80% G + C with Tm of 58–60 °C and the probe should have Tm approximately 10 °C higher: qEndoz570F 5′- TAGGCGGCTGCCTAAGTTGGATG-3′, qEndoz720R 5′-CGTCAGTGTCAGACCAGAGTGTC-3′ and probe qEndoz630p 5′-FAM-CCAAAACTGGGCAGCTAGAGTGCGGAAGAGGAGT-BHQ1–3′.

The system was run on Applied Biosystems® 7500 Real-Time PCR System using TaqMan® Fast Advanced Master Mix (Life Technologies) with 1–5 μl template in a reaction volume of 20 μl under standard reaction and cycling conditions. Results were linear across the tested range 10^2^–10^6^ copies per μl (r^2^ = 0.996), using quantified PCR product as standards. No cross-hybridisation was observed with other epitheliocystis agents, including *Ca*. Piscichlamydia, *Ca.* Similichlamydia and *Ca.* Ichthyocystis (Seth-Smith, *ISME J*, in press).

### Genomics

Three samples produced sequence for analysis: Dpd28cystMDA, extracted from a single micromanipulated cyst from a 28 dph larva, of which 1 μl was subject to multiple displacement amplification (MDA, Genomiphi V2 WGA, GE Life Sciences) then pooled with the remaining 19 μl extract; Dpd21lrvCNM, from a 21 dph larva, with host DNA depleted using NEBNext Microbiome kit (NEB) and the resulting DNA subject to MDA; Dpd28tailN, a tail section of an infected larva at 28 dph, subject to NEBNext Microbiome kit. Sequences were run on Illumina Miseq in 12-plex with 250 bp paired end reads following Nextera library creation. An additional 6-plex run was performed on Dpd28tailN and read data from the two runs were pooled. Raw sequencing reads were adaptor and quality trimmed and filtered using Trimmomatic version 0.32 49. *De novo* assembly was performed using SPAdes v 3.1.0 50 under default settings, which includes read error correction. Assemblies were tested using various K-mers in both single-cell mode and multi-cell mode^51^ and quality assessed using QUAST^52^ and CGAL^53^ (Tables S1 and S2). Single-cell mode produced better assemblies than multi-cell mode, giving 62 776 scaffolds comprising 39.3 Mb. Each scaffold was screened against the NCBInr database using blastx and screened for eukaryotic scaffolds using MEGAN5 54. These were removed leaving 49 903 scaffolds covering 31.0 Mb, representing 75.7% of reads. Removal of further scaffolds representing contaminating DNA used parameters given by SPAdes: coverage was plotted against scaffolds and used to define a cut-off of 40×. Additionally, scaffolds below 400 bp were discarded as being unlikely to provide valuable genomic information. Scaffolds were then ordered against the genome of *E. elysicola* type strain DSM22380 (JOJP00000000)^24^ using Abacas (http://abacas.sourceforge.net/) with manual correction in ACT^55^. Scaffolds matching this reference were designated “core” whereas those not matching “accessory”. Automated annotation using Prokka^56^ was manually checked against Prodigal gene prediction^57^ and reference strain annotation (Genbank accessions JOJP00000000 and AREW00000000) using Artemis^58^ and ACT. Ribosomal RNA operons were identified in multiple copy, adjusted and placed according to the type strain genome. Further *Endozoicomonas* genomes were also used for comparison (*E. montiporae* JOKG00000000 and *E. numazuensis* JOKH00000000 24). Mapping of raw and corrected reads to reference genomes and the assembly was performed using SMALT (http://sourceforge.net/projects/smalt/) and bwa^59^. Through mapping to the assembly, a number of heterozygous sites were found, which might indicate a mixed infection of two or more strains from the same species; our assembly and downstream analysis represent data from the dominant strain.

Raw read data for three samples sequenced has been submitted to the European Nucleotide Archive (ENA) with the accession numbers: Dpd28CystMDA ERR361041, and study PRJEB7440 for samples Dpd21lrvCNM and Dpd28tailN.

## Additional Information

**How to cite this article**: Katharios, P. *et al.* Environmental marine pathogen isolation using mesocosm culture of sharpsnout seabream: striking genomic and morphological features of novel *Endozoicomonas* sp. *Sci. Rep.* **5**, 17609; doi: 10.1038/srep17609 (2015).

## Supplementary Material

Supplementary Information

Supplementary Video S1

## Figures and Tables

**Table 1:** Probes used for FISH based on target bacterial 16S rRNA gene sequences^60^.

Probe	Specificity	Sequence/fluorophore	Position (*E. coli*numbering)	Reference
Chls-0523	Phylum Chlamydiae	5′-CCTCCGTATTACCGCAGC-3′Atto488	524–541	(Poppert *et al.*, 2002)
Endo-0474	*Ca.* E. cretensis	5′-AACCTTCAACCTTTCCTCCC-3′Cy3	471–490	This study
Endo-512	*Ca.* E. cretensis and *E. atrinae*	5′-GCTTCTTCTGTAGGTAACG-3′Cy5	509–528	This study
Dipvar-0410	Diverse chlamydial sequences amplified from larvae	5′-CCCGAAGGCCTTCTTCGCTCAC-3′Cy3	444–465	This study
Chls-0367	Phylum Chlamydiae	5′-GCTTTCGCCCATTGCGAA-3′Atto647	393–411	This study
