# Supplementary material for: Environmental marine pathogen isolation using mesocosm culture of sharpsnout seabream: striking genomic and morphological features of novel Endozoicomonas sp
Source: Sci Rep. 2015 Dec 7;5:17609. doi: 10.1038/srep17609 (PMC4671022; doi:10.1038/srep17609)
Supplement: Supplementary Information [file srep17609-s1.doc]

**Environmental marine pathogen isolation using mesocosm culture of sharpsnout seabream: striking genomic and morphological features of novel *Endozoicomonas* sp.**

Pantelis Katharios^1*^, Helena M.B. Seth-Smith^2,3*^, Alexander Fehr^3^, José M. Mateos^4^, Weihong Qi^2^, Denis Richter^3^, Lisbeth Nufer^3^, Maja Ruetten^3^, Maricruz Guevara Soto^3,5^, Urs Ziegler^4^, Nicholas R Thomson^6^, Ralph Schlapbach^2^, Lloyd Vaughan^3+^

1. Institute of Marine Biology, Biotechnology and Aquaculture, Hellenic Center for Marine Research, Heraklion, Crete, Greece

2. Functional Genomics Center Zürich, University of Zürich, Switzerland

3. Institute for Veterinary Pathology, Vetsuisse faculty, University of Zürich, Switzerland

4. Center for Microscopy and Image Analysis, University of Zürich, Switzerland

5. Center for Fish and Wild Animal Medicine, Vetsuisse Faculty, University of Bern, Switzerland.

6. The Wellcome Trust Sanger Institute, Hinxton, Cambridge, United Kingdom

*These authors contributed equally to this work

^+^ corresponding author

**Supplementary Information**

**Screening for Chlamydia – the false lead**

The pathogens initially sought in this study were those belonging to the phylum Chlamydiae, as these are the most commonly identified bacteria associated with epitheliocystis. The 280bp chlamydial signature sequence ^1,2^ is a useful screening tool for diverse members of the Chlamydiae, and was used to screen the larvae, plankton fractions and flow through water over the course of the mesocosm experiment (Figures S1 and S2). This provided intriguing molecular evidence for the presence of Chlamydiae in the water of the experimental tank from 15 dph, but not in the control tank from borehole water. At this stage, larvae showed no morphological signs of infection and were negative for Chlamydiae by signature PCR. The first positive PCR signal for Chlamydiae was found in the larvae at 21 dph, coinciding with the observation of the first epitheliocystis lesions in the fins of larvae from the experimental tank. This provided an initial indication that Chlamydiae were responsible for the pathology observed.

Subsequent to the experiment, longer Chlamydiae-specific 16S rRNA gene PCRs (see Methods) were used to screen DNA extracted from infected larvae from 20, 21 and 24 dph, and the fractionated plankton samples. Successful PCR, cloning and sequencing of these samples was challenging, leading to an initial indication that the responsible agents were more diverse and could not be identified with these standard techniques for Chlamydiae. The 16S rRNA gene sequences which were obtained are highly diverse and comprise members of a several news chlamydial clades (Figure S3). The sequences from the larvae form five clusters, and the sequences from the mesocosms form seven. The diversity seen did not fit with the concept of a single pathogen causing the epitheliocystis lesions, and there was little overlap between the larval and fraction sequences, indicating perhaps a sampling of environmental chlamydial diversity. One novel clade in particular contains over 20 new sequences with <90% identity to known Chlamydiae, and is likely to represent a new family ^3^. This clade is currently only represented by a handful of published uncultured bacteria identified only through 16S rRNA gene sequences (eg ^4-7^). An additional two clades were identified: one within the more distantly related *Ca*. Piscichlamydia clade, and one localised to the *Ca*. Simkania clade, the bulk of which originate from the plankton fractions 120-250 μm. This expands our knowledge of the diversity of chlamydial organisms in marine environments, but appears unconnected to epitheliocystis. The initial positive signal from the chlamydial signature PCR may have arisen from mis-priming against *Endozoicomonas* 16S rRNA gene sequences, as these primers produce an amplicon of the target size from *Endozoicomonas elysicola* genomic DNA (data not shown). The Chlamydiales qPCR system, however, does not detect *E. elysicola* DNA, and thus the results in Table S3 are specific.

Following histological assessment of the epitheliocysts, FISH was attempted to localise the novel chlamydial 16S rRNA gene sequences to the pathological lesions. Although cysts were evident in adjacent sections stained with HE, and were strongly labelled with DAPI, Chlamydiae-specific probe Chls-0523 did not result in any labelling of the cysts (data not shown). Two further probes were designed: Chls-0367 for the Chlamydiae phylum and Dipvar-0410 specifically to the chlamydial sequences amplified from the larvae. While Chls-0367 did produce signal, Dipvar-0410 gave no labelling of the cysts (Figure S4). Chls-0367 was later found to coincidentally only contain 2 mismatches to the *Endozoicomonas* 16S rRNA gene sequence over its 18bp length. Dipvar-0410_Cy3 gave faint signal, with 5 mismatches to the *Endozoicomonas* 16S rRNA sequence in this region. Results from FISH only gave consistent logical results once the pathogen had been identified as a species of *Endozoicomonas* and further appropriate probes designed (Figure S4). In the light of this information, explanations could be found for the success or otherwise of the previously tested FISH probes. Use of appropriate controls and multiple probes is critical when investigating novel pathogens, as is the screening of probes used against all putative pathogen sequences.

**
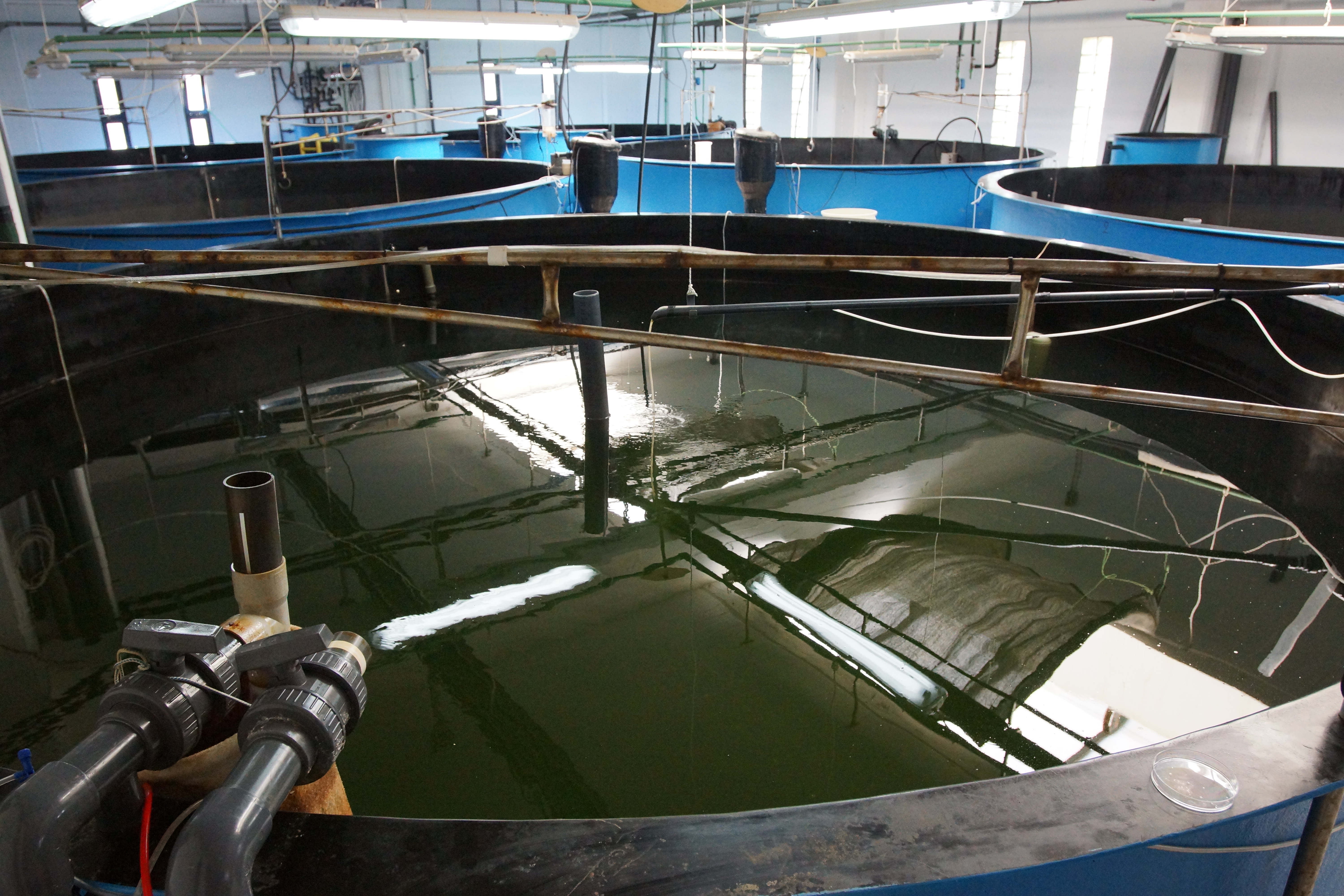
**

**Figure S1. Photograph of the mesocosm tanks during use.** Each tank has a diameter of 5m, a height of 2.35m and a working volume of 40m^3^ (40 000 litres). The green colour is from the microalgae *Chlorella minutissima*, added daily to the rearing water along with enriched rotifiers *Brachionus plicatilis* and enriched *Artemia* sp. nauplii (see Methods).


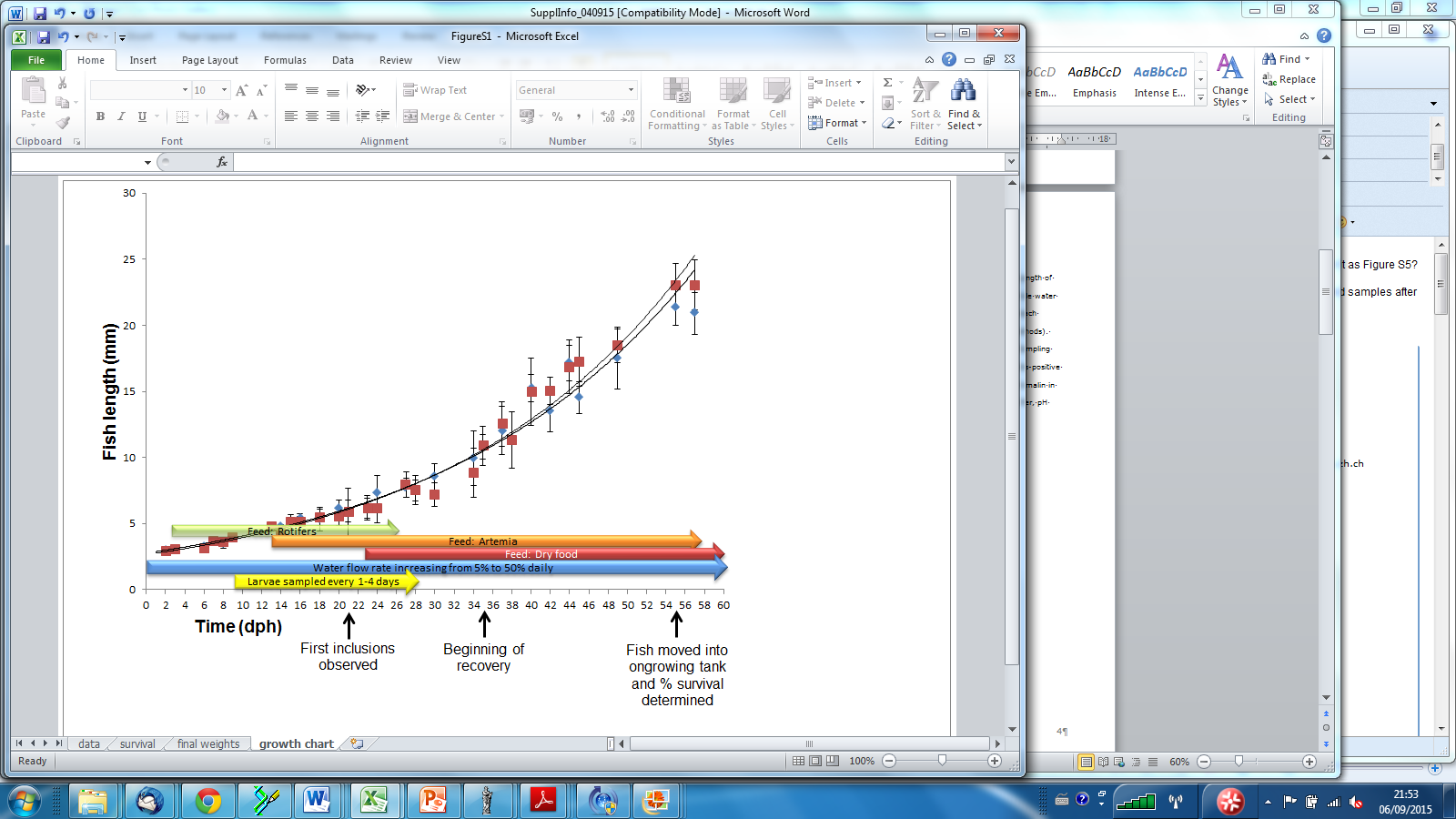


**Figure S2: Schematic of experimental set up and larval growth rates.** Length of larvae grown in the sea water mesocosm ( ) and those from the control borehole water mesocosm ( ). The mean and standard deviation of 10-15 measurements at each timepoint is plotted and analysed by regression analysis (see Materials and Methods). Superimposed is the feeding regime and the onset of infection and recovery. Sampling and diagnosis of 10-20 larvae was performed daily from 9-28 dph, and individual epitheliocystis-positive larvae, or microdissected cysts from these, were sampled into 10% buffered formalin in seawater; 4F:1G fixative; 2.5% glutaraldehyde in 0.1 M sodium phosphate buffer, pH 7.5; 70% ethanol; or RNALater as described in Methods.

**
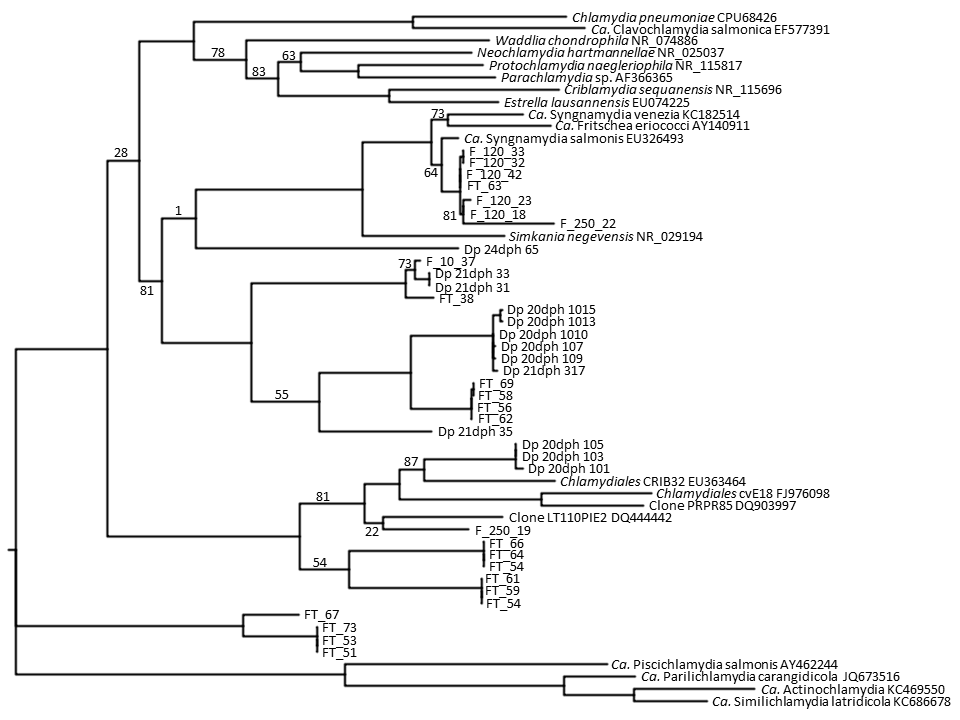
**

**Figure S3. Phylogenetic analysis of chlamydial sequences amplified from infected larvae, plankton fractions and flow through water.** Phylogeny of sequences longer than 1 kb, with reference sequences provided for a phylogenetic framework (EMBL accession numbers given). Where bootstrap values were under 90% they are indicated on branches (as %). Sequences derived from larval DNA extracts begin “Dp” with the sampling time post hatching (dph) and a laboratory clone code. Sequences derived from filtrate samples and flow through fractions (13 dph) are labelled “F” and the filter pore size, or “FT”, respectively, with a laboratory clone code. All novel sequences have been deposited with NCBI GenBank under the accession numbers KP857947-KP857983.

**
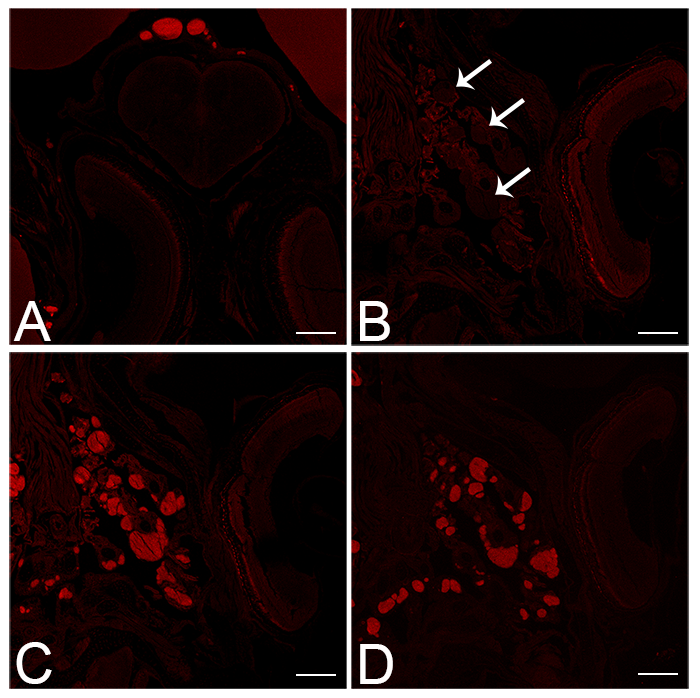
**

**Figure S4. FISH on 24 dph sharpsnout seabream larval sections with diverse probes. A.** Section probed with Chls-0367-Atto647 producing positive signal. **B.** Section probed with Dipvar-0410-Cy3 targeting amplified chlamydial sequences, with cysts not showing hybridisation (arrows). **C.** Section probed with Endo-0474-Cy3 against the novel *Endozoicomonas* 16S rRNA gene sequence showing strong hybridisation to cysts. **D.** Section probed with alternative probe Endo-0512-Cy5 against the novel *Endozoicomonas* 16S rRNA gene sequence showing strong hybridisation to cysts. All scale bars represent 100 μm.

**
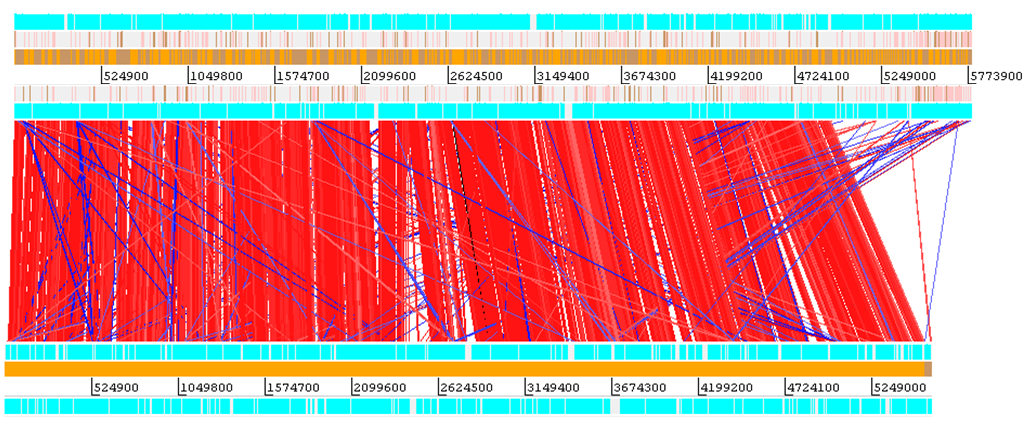
**

**Figure S5. Genome comparison of *Ca*. E. cretensis against the type strain *E. elysicola* DSM22380.** Draft genome scaffolds of *Ca*. E. cretensis (top) were ordered against that of *E. elysicola* DSM22380 (bottom), compared using blastn and visualised using ACT ^8^. Deep red bars indicate high levels of nucleotide identity (>90%) in the core regions of the genome of this genus. Blue bars indicate inversions, and paler colours show lower nucleotide identity. Areas where no matches are visible indicate insertions or deletions in one genome relative to the other. Orange and brown bars indicate scaffolds along the draft genomes, turquoise bars above and below this indicate putative coding sequences (CDSs) in forward and reverse frames. For *Ca.* E. cretensis, pink and brown bars in the separate tracks represent transposases, intact and pseudogenes respectively, in forward and reverse frames. Smaller scaffolds on the right indicate putative accessory scaffolds of *Ca*. E. cretensis.

| **Assembly** | **single-cell, contigs** | **single-cell, scaffolds** | **multi-cell, contigs** | **multi-cell, scaffolds** |
| --- | --- | --- | --- | --- |
| # contigs (>= 0 bp) | 62791 | 62776 | 39201 | 39205 |
| # contigs (>= 1000 bp) | 4739 | 4734 | 4297 | 4293 |
| Total length (>= 0 bp) | 39315503 | 39315042 | 30881129 | 30886171 |
| Total length (>= 1000 bp) | 12697269 | 12699876 | 12375260 | 12376698 |
| # contigs | 21787 | 21783 | 20054 | 20051 |
| Largest contig | 91550 | 91550 | 134527 | 134527 |
| Total length | 23924425 | 23927316 | 22711333 | 22713434 |
| GC (%) | 46.69 | 46.69 | 46.96 | 46.96 |
| N50 | 1085 | 1085 | 1140 | 1140 |
| N75 | 668 | 668 | 671 | 671 |
| L50 | 4033 | 4027 | 3341 | 3337 |
| L75 | 11325 | 11319 | 10127 | 10123 |
| # Ns per 100 kbp | 0.02 | 0.76 | 0 | 3.85 |

**Table S1. Genome assembly metrics through QUAST.** Comparing SPAdes single-cell and multi-cell modes for the initial two assemblies.

| **Assembly** | **# contigs** | **Total likelihood** | **Likelihood mapped reads** | **Likelihood unmapped reads** | **Total # PE reads** | **# unmapped reads** |
| --- | --- | --- | --- | --- | --- | --- |
| sc, contigs | 62791 | -452407539 | -96440895 | -355966644 | 3077515 | 778219 |
| mc, contigs | 39201 | -491659667 | -91184649 | -400475018 | 3077515 | 803933 |

**Table S2. Genome assembly likelihood through CGAL.** The higher the total likelihood, the better the assembly. Contig metrics were used for this analysis. Sc, single-cell mode; mc, multi-cell mode.

|  |  |  | ***Endozoicomonas* specific qPCR** | | **Chlamydiae specific qPCR** | |
| --- | --- | --- | --- | --- | --- | --- |
|  |  |  |  | mean of all larvae |  | mean of all larvae |
|  |  |  | Mean per larva ± sd |  | Mean per larva ± sd |  |
| Experimental mesocosm | Larvae 21 dph | 1 | 15333333 ± 4041452 |  | 8100 ± 566 |  |
| sea water |  | 2 | 36666667 ± 2516611 |  | 51050 ± 17607 |  |
|  |  | 3 | 9766667 ± 2200757 |  | 14150 ± 9829 |  |
|  |  | 4 | 12766667 ± 3043572 |  | 75300 ± 28567 |  |
|  |  | 5 | 14766667 ± 5353815 | 17860000 | 2850 ± 1768 | 30290 |
| Experimental mesocosm | Larvae 24 dph | 1 | 160000000 ± 20000000 |  | 25000 ± 11172 |  |
| sea water |  | 2 | 70000000 ± 17691806 |  | 15150 ± 19870 |  |
|  |  | 3 | 107000000 ± 24637370 |  | 13800 ± 4808 |  |
|  |  | 4 | 85000000 ± 11532563 |  | 3400 ± 1980 |  |
|  |  | 5 | 143333333 ± 5773503 | 113066667 | 7200 ± 1556 | 12910 |
|  |  |  | Mean per μl ± sd |  | Mean per μl ± sd |  |
| Experimental mesocosm | Fractions (mm) | 10 | 0 |  | 0 ± 0 |  |
| sea water |  | 25 | 0 |  | 423 ± 18 |  |
|  |  | 50 | 0 |  | 426 ± 54 |  |
|  |  | 120 | 0 |  | 21 ± 9 |  |
|  |  | 250 | 390 ± 28 |  | 643 ± 42 |  |
| Control mesocosm | Fractions (mm) | 10 | 0 |  | 2390 ± 99 |  |
| borehole water |  | 25 | 0 |  | 8350 ± 552 |  |
|  |  | 50 | 0 |  | 4215 ± 1520 |  |
|  |  | 120 | 0 |  | 1097 ± 330 |  |
|  |  | 250 | 0 |  | 371 ± 144 |  |

**Table S3. Load of *Endozoicomonas* and Chlamydiae in infected larvae and mesocosm water.** Values were determined by qPCR, measured in triplicate for the *Endozoicomonas* target and in duplicate for chlamydial target. Values are given as mean copy number of 16S rRNA gene target per larva or per μl extract, with standard deviation. More copies of *Endozoicomonas* 16S rRNA gene can be amplified from larvae 24 dph than 21 dph, with lower levels of Chlamydiae present at 24 dph. *Endozoicomonas* was not identified in plankton fractions of either experimental or control mesocosm water at 13 dph (the 250 μm fraction is likely to have contained some infected larvae). Chlamydiae can be detected in the sea water fractions of both the experimental and control mesocosms, more so in the borehole water, perhaps carried opportunistically in plankton hosts.

| **Sample** | ***E. elysicola* type strain genome mapped (%)** | **Mean coverage** |
| --- | --- | --- |
| Dpd28cystMDA | 73.3 | 8.0 x |
| Dpd28tailN | 75.3 | 63.0 x |
| Dpd21lrvCNM | 78.1 | 59.5 x |

**Table S4. Mapping statistics of samples sequenced from infected sharpsnout seabream tissue.**

**Video S1: Video of two neighbouring cysts from a larva 24 dph obtained by FIB-SEM.** The bacteria are lined up along the cyst membrane, often in the same orientation. Bacteria contain paler and darker material and pale, possibly lipid-filled, vacuoles. Segmentation analysis of the 3D data created a rendered model of an apparently dividing bacterium with many vacuoles inside and the surrounding filament network.

**Supplementary References**

1 Everett, K. D. E., Bush, R. M. & Andersen, A. A. Emended description of the order Chlamydiales, proposal of *Parachlamydiaceae* fam. nov. and *Simkaniaceae* fam. nov., each containing one monotypic genus, revised taxonomy of the family *Chlamydiaceae*, including a new genus and five new species, and standards for the identification of organisms. *Int J Syst Bacteriol* **49**, 415-440 (1999).

2 Polkinghorne, A., Schmidt-Posthaus, H., Meijer, A., Lehner, A. & Vaughan, L. Novel *Chlamydiales* associated with epitheliocystis in a leopard shark *Triakis semifasciata*. *Dis Aquat Org* **91**, 75-81, doi:10.3354/dao02255 (2010).

3 Everett, K. D. E., Hornung, L. J. & Andersen, A. A. Rapid Detection of the *Chlamydiaceae* and Other Families in the Order *Chlamydiales*: Three PCR Tests. *J Clin Microbiol* **37**, 575-580 (1999).

4 Corsaro, D. *et al.* Novel *Chlamydiales* strains isolated from a water treatment plant. *Environ Microbiol* **11**, 188-200, doi:10.1111/j.1462-2920.2008.01752.x (2009).

5 Schubert, C. J. *et al.* Anaerobic ammonium oxidation in a tropical freshwater system (Lake Tanganyika). *Environ Microbiol* **8**, 1857-1863, doi:10.1111/j.1462-2920.2006.001074.x (2006).

6 Corsaro, D. & Venditti, D. Detection of Chlamydiae from freshwater environments by PCR, amoeba coculture and mixed coculture. *Res Microbiol* **160**, 547-552 (2009).

7 Zhu, P., Li, Q. & Wang, G. Unique microbial signatures of the alien Hawaiian marine sponge Suberites zeteki. *Microb Ecol* **55**, 406-414, doi:10.1007/s00248-007-9285-3 (2008).

8 Carver, T. J. *et al.* ACT: the Artemis Comparison Tool. *Bioinformatics* **21**, 3422-3423, doi:10.1093/bioinformatics/bti553 (2005).
